# Multimodal Artificial Intelligence for the Clinical Assessment of Otitis Media

**DOI:** 10.3390/jcm15218405

**Published:** 2026-10-28

**Authors:** Jae Ho Chung, Yoonseob Lim, Hee Won Seo, Sang-Yoon Han

**Affiliations:** 1 Department of Otolaryngology-Head and Neck Surgery, College of Medicine, Hanyang University, Seoul 04763, Republic of Korea; 2 Department of HY-KIST Bio-Convergence, College of Medicine, Hanyang University, Seoul 04763, Republic of Korea; 3 Korea Institute of Science and Technology (KIST), Seoul 02792, Republic of Korea

**Keywords:** otitis media, otoscopy, multimodal artificial intelligence, tympanometry, pure-tone audiometry, diagnostic uncertainty, large language models

## Abstract

Acute otitis media (AOM) and otitis media with effusion (OME) can often be recognized from characteristic otoscopic findings. However, patients also present with subtle, overlapping, or resolving findings that are difficult to assign to a single diagnosis from an image alone. If these encounters are excluded because the reference diagnosis is uncertain, artificial intelligence (AI) models may perform well on selected images while providing less help in clinically uncertain cases. This focused narrative review examines whether symptoms, tympanometry, and hearing information can complement otoscopic images and improve the representation of clinically uncertain middle ear disease. Selected studies have combined images with acoustic measurements, pure-tone audiometry, or patient information. Their findings support feasibility, but differences in diagnostic targets and study design limit conclusions about benefit in uncertain AOM and OME. We propose that future systems account for diagnostically challenging cases during development and include them in clinical evaluation, while describing the current ear status alongside any diagnostic label. Large language models may help express supported findings, their clinical interpretation, and remaining uncertainty in readable language. Such explanations require evaluation independent of classification accuracy. The clinical value of multimodal AI should be established by its ability to support appropriate decisions in representative patients, including those with incomplete or discordant findings.

## 1. Introduction

Otoscopy is central to the diagnosis of otitis media. In many patients, characteristic findings make acute otitis media (AOM) or otitis media with effusion (OME) readily recognizable. Nevertheless, the interpretation of a tympanic membrane image depends on the clinical question. Identifying fluid behind the membrane, recognizing acute inflammation, and determining the hearing consequences of middle ear disease require related but different information. Diagnostic guidance therefore considers otoscopic findings together with symptoms and other examination findings [1,2].

Artificial intelligence (AI) has shown encouraging performance in the classification of otoscopic images. Reviews have summarized this work across several ear diseases [3,4]. A video-based AOM model, for example, reported sensitivity of 93.8% and specificity of 93.5% in internal validation [5]. Seo et al. developed an image-based pipeline that assessed image quality, segmented the tympanic membrane, determined laterality, and classified normal ears, AOM, OME, and chronic otitis media. In a retrospective dataset of 2964 images, the reported disease-classification accuracy was 88.66%, with F1-scores of 0.78 for AOM and 0.87 for OME [6]. These results support the value of visual information. They also raise a practical question: how well will an image model perform when the appearance is less typical, the history changes its interpretation, or the diagnosis remains uncertain after examination?

This question matters because patients do not always present with a fully developed or stable pattern. An ear may show mild erythema, an amber effusion, an air–fluid level, or residual abnormalities after acute symptoms have improved. An otoscopic image cannot establish the onset of symptoms or describe the patient’s hearing. When such cases do not receive an agreed reference label, investigators may exclude them from model development. We hypothesize that this could leave a gap between the cases that an AI system learns to classify and the cases for which clinicians need assistance.

Multimodal AI provides a way to examine this gap. Symptoms and history can provide the temporal and inflammatory context of the image. Tympanometry can contribute evidence about middle ear function, and hearing assessment can describe functional impairment when an appropriate test is feasible. The purpose of combining these data is to represent the patient’s condition more completely. Whether this improves diagnosis must be demonstrated, particularly in patients whose initial findings are inconclusive.

This review examines the clinical reasons for combining these sources of information, the studies that have attempted such integration, and the limitations of their evidence. We also consider a further development: an AI output that explains the current ear status instead of providing only a normal, AOM, or OME label. We propose a clinical framework for this approach and identify the evidence needed to establish its usefulness.

## 2. Review Scope

This focused narrative review examines whether information obtained in addition to otoscopy can improve the assessment of middle ear disease, particularly when the initial findings are inconclusive. We prioritized original human studies that combined an otoscopic image or video with a functional measurement or clinical information from the same patient and reported a diagnostic result. Studies of broader middle ear disorders were eligible when they informed the proposed approach, but their findings were not treated as direct evidence for AOM–OME differentiation. Image-only studies, clinical guidelines, and reporting standards were used as supporting literature.

A targeted PubMed search was conducted on 1 October 2026, along with supplementary source checking to identify relevant studies. A total of seven studies were selected for the core comparison. Appendix A provides the detailed search strategy, eligibility criteria, and literature selection process.

## 3. When Otoscopic Images Do Not Tell the Whole Story

### 3.1. Typical Findings and Changing Clinical States

The distinction between AOM and OME is often clear when the findings are typical and the clinical information is available. However, membrane redness, opacity, bulging, and visible fluid do not always appear together or change at the same time. Mild redness alone does not establish AOM. Similarly, a visible effusion does not establish that the patient has an acute symptomatic inflammatory episode. OME is defined by middle ear fluid without the acute manifestations that characterize AOM [1,2].

Normal ears, AOM, and OME should therefore not be treated as obligatory stages in a single linear sequence. Their relevant findings can overlap and change during illness and recovery. Effusion may remain after an acute episode has improved. Other patients may have effusion without a recognized preceding episode of AOM. An amber-colored tympanic membrane or air–fluid level describes an observation; the diagnosis also depends on the other findings and their timing. This is the sense in which we consider middle ear disease to have continuous and changing clinical features.

The difficulty is measurable. Sundgaard et al. collected 1409 cases, including 204 assessed by all four otolaryngologists. After six indeterminate cases were excluded from this main analysis, complete agreement was 57%, with a Light’s kappa of 0.58. Agreement was higher for AOM. Adding Wideband tympanometry (WBT) increased diagnostic certainty for OME and no-effusion cases, but the increase in inter-rater agreement was not statistically significant [7]. Reader disagreement does not identify the correct diagnosis, but it illustrates the difficulty of assigning a single image-based label.

### 3.2. Diagnostic Uncertainty and Case Selection in AI

Supervised learning requires a reference target, commonly called ground truth. Clear images with an agreed diagnosis are easier to label and use for training. The selection of cases for AI development should depend on the intended diagnostic task. While excluding poor-quality images or conditions outside the target categories may be appropriate, excluding diagnostically uncertain cases within the intended clinical scope may limit the model’s applicability to challenging presentations.

Kim et al. excluded 152 of 1632 records (9.3%) for reasons that included severe swelling, bleeding, indistinguishable disease, and overlapping or blurred image focus [8]. These mixed reasons do not allow the proportion excluded specifically for diagnostic uncertainty to be calculated. Noda et al. excluded poor-quality images, postoperative cases, and cases with multiple suspected diseases, but did not provide a separate count for uncertainty [9]. The six indeterminate cases among the 204 examinations assessed by four readers in Sundgaard et al. (2.9%) describe a reader-study subset, not the prevalence of uncertainty in AI training datasets [7].

Byun et al. used a platform requiring no coding experience to develop an image classifier from 3024 tympanic membrane images. On a separate set of 80 representative images, overall accuracy was 78.75%, and OME was often mistaken for a normal membrane [10]. Poor-quality images were excluded, and AOM was not included as a diagnostic category. Clinical records and audiologic findings informed the reference labels, but the model itself received only images. This illustrates the distinction between clinical information used to establish a diagnosis and information actually available to the model.

There are also examples of investigators addressing difficult cases directly. Song et al. combined tympanic membrane images and Pure-tone audiometry (PTA) in an anomaly-detection approach. Their evaluation included 35 records with coexisting diseases and 81 records described as requiring treatment despite difficulty assigning a specific diagnosis [11]. Sundgaard et al. jointly predicted diagnosis and diagnostic difficulty [12]. These studies argue against a general claim that difficult cases are excluded from AI research. Our concern is narrower: exclusion may limit a model’s usefulness when the excluded encounters resemble the patients for whom assistance is most needed. The frequency and clinical effect of this selection remain to be established.

Retaining a difficult case does not mean forcing a diagnosis. AI development and evaluation should consider diagnostically challenging cases to better reflect the complexity of real-world clinical practice. In such cases, the final diagnosis may remain indeterminate. Subsequent examinations may clarify the course, but future information must be kept separate from the inputs available at the initial encounter. This approach would allow AI systems to account for diagnostic uncertainty without treating an unsupported categorical label as established fact.

### 3.3. Establishing a Reference Diagnosis in Uncertain Cases

AOM and OME do not have a single independent reference test that captures every clinically relevant feature. Myringotomy can confirm fluid in the selected patients who undergo surgery, but fluid alone does not determine whether the current episode is AOM or OME. Expert review of an image assesses morphology, while a clinical assessment can additionally consider symptom onset and examination findings. The appropriate reference therefore depends on what the model is intended to estimate.

A composite clinical diagnosis is reasonable when the target is the patient’s clinical condition. However, if symptoms, images, and tympanometry contribute both to the model and to its reference diagnosis, the evaluation may favor a model that reproduces the information used to establish the label. This dependence, including possible incorporation bias, should be reported. Blinding the reference assessors to the AI output is necessary but does not remove the dependence created by shared clinical inputs.

The practical response is to make the reference process explicit. Studies should specify the diagnostic criteria, the information available to reviewers, and how disagreement was handled. They should preserve the underlying observations as well as the final diagnosis. A system may correctly identify evidence of effusion while remaining uncertain about acute inflammation. Evaluating these outputs separately provides more information than assigning the entire encounter a single correct or incorrect label.

The study-specific reference procedures are summarized in Section 5. WBT contributed to the reference diagnosis in the paired image–WBT study, whereas Lu et al. assigned video-based reference labels without access to tympanometry or AI predictions [12,13]. The latter reduces incorporation of tympanometry, but a visual reference still does not capture every clinical aspect of disease. Where feasible, sensitivity analyses using a reference assessment without the added modality can help examine how shared information affects the comparison.

Follow-up findings may provide a separate outcome when the clinical state becomes clearer over time. They should not be entered as information available at the initial visit. Surgical findings can establish the presence of effusion in selected patients, but cannot serve as a reference for all outpatient encounters. An indeterminate reference should remain identifiable rather than being converted into an apparently certain label.

## 4. What Symptoms and Hearing Tests Add

### 4.1. Symptoms and the Course of Illness

Symptoms help determine what an otoscopic finding means at the time of assessment. Recent ear pain, the duration of symptoms, prior treatment, and the direction of clinical change can alter the interpretation of an abnormal membrane. A similar photograph may occur during an acute presentation or after symptoms have subsided. These details should be part of the initial clinical assessment, rather than information requested only after an image model becomes uncertain.

Clinical data also have limitations. Symptoms may be nonspecific, young children may not report them reliably, and an unrecorded symptom is not equivalent to its absence. Models should distinguish a negative response from missing information. They should use the observations available at the intended decision point and avoid treating a previously entered diagnosis or treatment decision as if it were independent evidence. The purpose is to learn the relationship between the examination and the history, while preserving the limitations of both.

### 4.2. Tympanometry

Tympanometry examines the acoustic response of the middle ear and can supplement an uncertain otoscopic assessment. Its interpretation depends on the tracing, ear canal volume, examination quality, and clinical setting. An abnormal response does not by itself distinguish AOM from OME or establish a need for antibiotics. Wideband acoustic immittance (WAI), including wideband tympanometry, provides measurements across frequencies and, when obtained under varying pressure, across pressures. It offers a broader description of mechanical behavior than a single summary curve.

Component studies support the information contained in acoustic measurements. Grais et al. examined AI classification of normal and OME ears using WAI, while Merchant and Neely examined the relationship between wideband measurements and effusion volume [14,15]. A WBT model reported 92.6% accuracy for otitis media detection but could not reliably distinguish AOM from OME [16]. This finding is clinically understandable: mechanical abnormality and acute inflammatory disease are related but distinct targets.

Longitudinal measurements may also help describe change. A study published in the 2025 volume of the Journal of Audiology and Otology evaluated WBT findings during recovery from OME [17]. It supports the relevance of repeated functional assessment, although recovery was defined using tympanometry and the study did not test a multimodal AI system. Such data could inform future models of clinical course, provided that repeated measurements and their timing are handled appropriately.

### 4.3. Hearing Assessment When Feasible

When feasible, hearing assessment addresses a clinically important but distinct question that an otoscopic image cannot answer: the functional consequence of the middle ear condition. PTA can characterize hearing thresholds and a conductive component in patients who can complete testing reliably. Its main role in the proposed framework is to describe hearing impact, while otoscopy, symptoms, and tympanometry support the assessment of the middle ear condition. PTA does not independently distinguish AOM from OME.

Conventional PTA is not a required input, particularly in young children. Age-appropriate testing should follow the clinical indication, and the system should remain usable when hearing results are unavailable [2]. Reliable measurements are essential; Cho et al. describe relevant audiometric calibration standards [18].

Table 1 summarizes the questions addressed by each source of information. The inputs may agree, or they may leave a clinically meaningful discrepancy. An explanation should preserve that discrepancy rather than averaging it into an apparently certain diagnosis.

## 5. What Multimodal Studies Have Shown

Multimodal AI has been investigated in otoscopic diagnosis using various combinations of imaging, functional measurements, and clinical information. The available studies address different diagnostic targets, including AOM, OME, and other middle ear disorders, providing insights into different approaches to multimodal integration. Multimodal approaches differ in where they combine information. Early fusion brings input variables together before separate representations are learned. Intermediate fusion combines features extracted from each modality. Late fusion combines predictions or decisions. We use these operational definitions because terminology differs between studies. For example, Kim et al. describe their approach as late fusion, but the reported attention, concatenation, and gating operate on learned features; we classify it here as intermediate feature fusion [8]. The image–WBT model also combines learned representations [12]. Binol and Stephens combine image-derived predictions with tympanometry [19,20].

Confidence-guided fusion is a sequential decision strategy in which an additional input modifies an uncertain initial prediction [13]. It does not necessarily decide whether a new test should be acquired. Prompting a pretrained vision-language model with an image and patient information is different from training a task-specific model on paired records [9]. Using an Large language model (LLM) to explain an existing prediction is a further, separate role [21]. Early fusion is included to define the terminology; we did not identify a clear example of raw-input early fusion among the seven selected studies.

### 5.1. Combining Otoscopic Images with Tympanometry

The paired image and tympanometry studies provide the most direct evidence for adding functional measurements to otoscopy. Binol et al. reported an early decision-fusion study involving 73 paired records. Stephens et al. subsequently evaluated combined information in 813 children from remote communities. Both reported higher classification accuracy with combined inputs, as summarized in Table 2 [19,20]. Both studies evaluated broad abnormality detection. Their results do not establish the added value for distinguishing AOM from OME.

Sundgaard et al. evaluated joint otoscopic image and WBT inputs in 1014 paired observations. The study also considered diagnostic difficulty, making it relevant to uncertain cases. The combined model showed a modest numerical gain over images alone, including an increase in the AOM F1 score; the image-only versus combined accuracy difference was not statistically significant. The reference diagnosis incorporated WBT, which limits interpretation of the added modality’s independent contribution [12].

Lu et al. used tympanometry to revise low-confidence video classifications. Their adult external paired cohort showed a larger numerical improvement, but the reported best threshold was selected in that evaluation cohort. The six-category task did not include AOM. The study therefore supports further testing of selective decision fusion, rather than establishing that this strategy resolves uncertain AOM versus OME or determines when an additional examination should be performed prospectively [13].

### 5.2. Combining Otoscopic Images with Pure Tone Audiometry

Kim et al. trained a model using paired tympanic membrane images and PTA data. The five target categories included normal ears, perforation, retraction, cholesteatoma, and a normal membrane with hearing loss. The study demonstrates that imaging and hearing information can be combined, but it did not evaluate AOM or OME. Its image-only comparison also used a different number of classes, so the reported performance cannot establish a matched incremental benefit for otitis media diagnosis [8].

Song et al. examined a broader question: whether images and hearing measurements could identify an abnormal ear without assigning a specific disease label [11]. Their single-center study included 1470 paired records. The Cooperative Observation Network was trained on 271 normal records and tested on 1199 records, comprising 68 normal and 1131 abnormal records. Image features and two PTA measures, mean air-conduction threshold and air–bone gap, were combined through cross-attention before anomaly classification. The model achieved 96.75% accuracy and a reported AUC of 0.93. It correctly classified 1099 of 1131 abnormal records (97.17%) and 61 of 68 normal records (89.71%). The abnormal group included 218 records with a normal-appearing membrane and hearing loss, as well as coexisting and difficult-to-classify disorders.

The study also compared the combined model with versions using only images or only PTA under the same reported data and learning-rate conditions. Both single-input versions classified every record as normal, giving 5.67% accuracy and an AUC of 0.50, compared with 96.75% and 0.93 for the combined model [11]. This supports the contribution of both inputs within this architecture. However, the failure of both single-input versions to distinguish the classes limits conclusions about the benefit over an effective image-only comparator. The test set was predominantly abnormal, external validation was not reported, and patient-level separation and reference adjudication were insufficiently described. The study supports broader assessment of ear abnormalities, but does not establish improved AOM–OME differentiation.

This distinction is important for future research. Adding hearing information can change both the available evidence and the definition of the target. A hearing-loss category partly defined by audiometry will naturally depend on audiometric information. For an AOM–OME study, investigators should prespecify whether PTA is intended to improve disease classification, describe functional consequences, or both. Those purposes require separate outcome measures.

### 5.3. Combining Otoscopic Images with Patient Information

Noda et al. gave GPT-4 Vision otoscopic images together with age, sex, and chief complaints. They developed prompts using 40 images and tested a separate set of 190 images covering four diseases. Accuracy was 82.1%. The model was already pretrained; it was not trained on the paired clinical records used in this study. The study did not quantify the added benefit of patient information in a matched comparison, and normal ears were not included [9].

The study nevertheless addresses a clinically relevant direction: the image is interpreted in the context of the patient. Future work should establish when that context corrects an image-based error and when it creates an error. A system could overemphasize a nonspecific symptom or give insufficient weight to a convincing examination. Evaluating these changes case by case is more informative than reporting only an overall accuracy difference.

The reference diagnosis procedures and the size and independence of the evaluation are important (Table 3). Binol provides a small conference study [19]. Stephens includes 813 children from several communities, but repeated frames are not independent patients, and the reported split does not clearly establish separation by patient and ear [20]. Sundgaard directly addresses AOM and OME but uses a single-clinic dataset and a reference that includes WBT [12]. Lu includes an external adult cohort, although selection of the best threshold in that cohort requires independent confirmation [13]. Kim, Noda, and Song demonstrate different ways to combine information, but do not establish a matched improvement in uncertain AOM–OME assessment [8,9,11].

### 5.4. What the Evidence Does Not Yet Establish

The selected studies support the feasibility of integrating visual, functional, or clinical information. They do not yet establish that a multimodal system closes the diagnostic gap in consecutive patients with uncertain AOM or OME. Some studies address broader middle ear abnormalities. Others use selected diagnostic groups, differing reference procedures, or comparisons that do not isolate the added information. Combining model outputs, jointly learning from paired measurements, and prompting a general model are also different interventions.

The most relevant next comparison is therefore performance in the same patients, using the same diagnostic target and reference procedure, with and without the additional information. Results should be reported separately for typical and uncertain presentations. The uncertain group should be defined before examining the model’s errors, using clinical criteria or reviewer disagreement. Otherwise, a favorable overall result may mainly reflect good performance in straightforward cases while leaving the intended clinical problem unresolved.

## 6. Explaining the Current Ear Status

### 6.1. Information Beyond a Diagnostic Label

Normal, AOM, and OME remain useful clinical categories. However, a label alone cannot show which findings are present, why they support that interpretation, or what remains unknown. For an uncertain encounter, a more useful output may describe the membrane, evidence of effusion, symptoms and their timing, middle ear function, and hearing information when available. The diagnosis can then be presented with its supporting evidence and limitations.

Consider an illustrative encounter with an amber effusion and an air–fluid level, a flat tympanogram with an appropriate ear canal volume, reliable testing showing a mild conductive hearing loss, and resolved pain after a documented recent episode of AOM. A proposed report might state: “The image and tympanogram support persistent middle ear effusion. Audiometry shows a mild conductive hearing loss. The supplied history indicates that the acute pain has resolved. Taken together, these findings are compatible with residual effusion after the recent episode. The available information does not establish ongoing acute inflammation.” This is an author-created example, not an output validated in a clinical study.

The report should change when the evidence changes. If symptom timing is missing, it should say that the acute clinical context cannot be determined. If image quality is inadequate, it should not claim that bulging or fluid is absent. If hearing was not tested, it should not infer a threshold from the diagnosis. Each statement should be traceable to a supplied observation or clearly identified as a model estimate. This provides a way to describe uncertainty without leaving the clinician with an unexplained low-confidence label.

**Figure 1:**
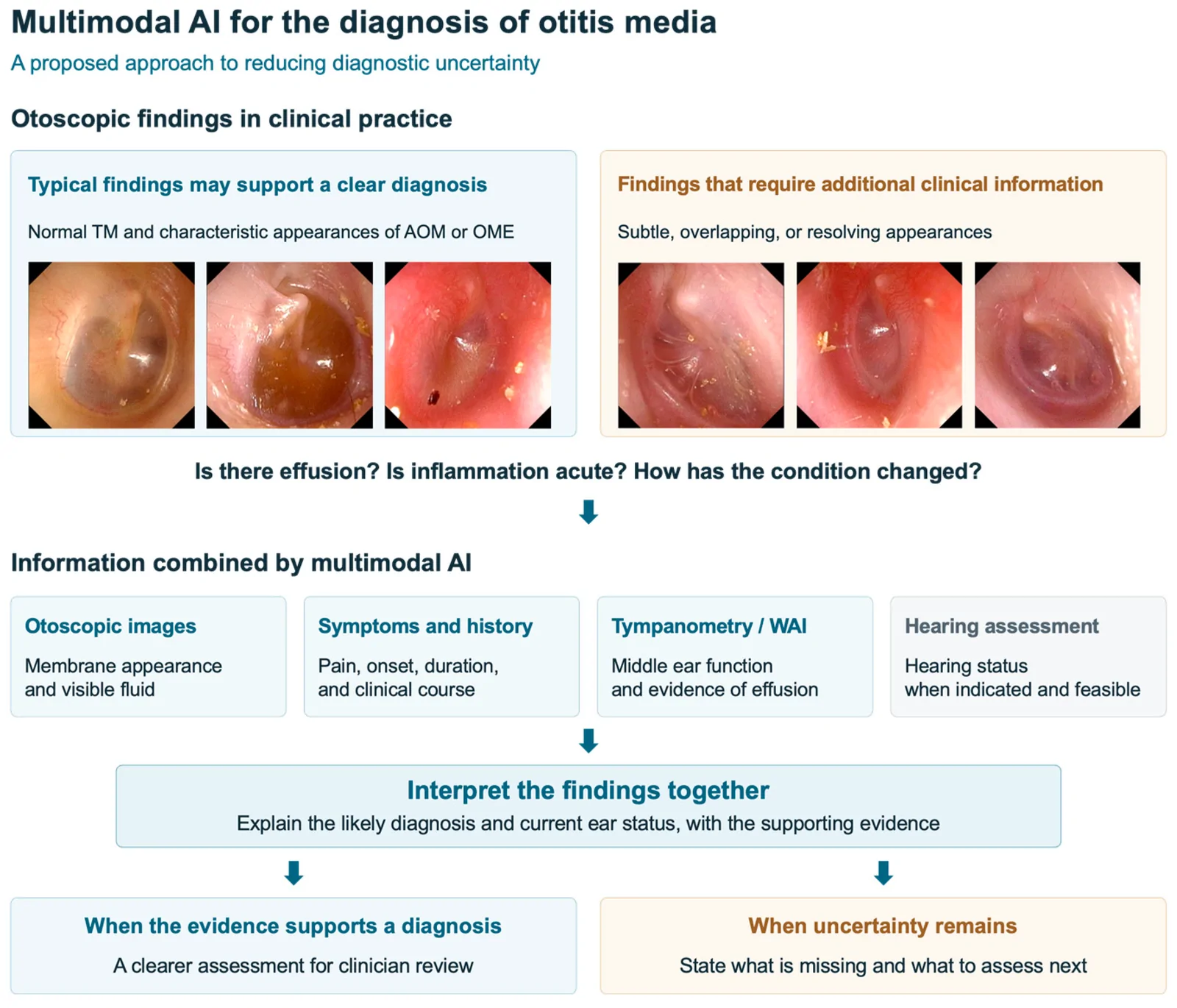
Proposed multimodal assessment of clinically uncertain otitis media. Development and evaluation retain typical, overlapping, and resolving presentations. Linked images, history, acoustic measurements, and hearing information when available support an account of the current ear status. The report preserves missing information and disagreement. An LLM may assist expression, but each statement requires support from the supplied data. This conceptual framework is not a validated diagnostic pathway and does not imply a fixed normal–AOM–OME sequence.

Figure 1 illustrates the proposed conceptual framework for integrating multimodal findings to describe the current ear status while preserving uncertainty, missing information, and disagreement.

### 6.2. The Potential Role of Large Language Models

Large language models (LLMs) may help convert structured findings into a readable clinical explanation. A vision-language model may also interpret images and text together. These roles should be distinguished. Generating a report from previously extracted findings is different from determining the findings directly, and an improvement in report readability does not establish an improvement in diagnosis.

Chu et al. described a hybrid system linking a convolutional neural network to LLM-based interpretation. In the reported workflow, the language model explained the classifier output without overriding its predictions. The study supports the feasibility of adding a language interface, but the classifier’s reported performance should not be attributed to the explanation stage. It also does not establish that generated explanations improve decisions in uncertain AOM or OME [21].

An LLM cannot resolve an uncertain reference diagnosis merely by producing a convincing paragraph. Training and evaluation still require examples that distinguish observed findings, clinical interpretation, and unresolved questions. Clinician-authored descriptions could provide supervision, including descriptions of ambiguous encounters. However, such descriptions should undergo review, and disagreements should be retained rather than concealed by selecting the most fluent account. This proposed approach requires prospective validation.

### 6.3. Evaluating the Explanation

The explanation should be assessed against the actual input record as well as the reference diagnosis. Reviewers should determine whether it accurately describes the visible and measured findings, uses the history appropriately, and identifies relevant missing information. Unsupported claims, such as an invented symptom or an unmeasured hearing deficit, should count as errors even if the final diagnosis is correct. Failure to express a clinically important discrepancy should also be recorded.

The clinical evaluation must then ask whether the explanation helps its intended reader. Useful outcomes include recognition of uncertainty, appropriate requests for further assessment, correction of diagnostic errors, and avoidance of inappropriate reassurance. The report should make a mistaken conclusion easier to recognize, rather than making it more persuasive. An apparently natural explanation is valuable only when its content is faithful and its effect on clinical judgment is beneficial.

## 7. What Studies Need to Show Before Clinical Use

### 7.1. Including the Patients Seen in Routine Practice

Prospective studies should enroll patients across the intended clinical spectrum and document all attempted examinations. They should retain borderline, resolving, and discordant presentations in the evaluation, including cases for which a final diagnosis remains uncertain. Technical failures and missing modalities should be reported as clinical outcomes of system use rather than silently removed from the denominator. A model that performs well only after successful acquisition of every test may have limited practical coverage.

The data should preserve the patient, ear, and visit relationships. Images from the same patient, bilateral ears, or repeated examinations should not cross training and test partitions in a way that permits information leakage. Follow-up observations can inform a separately defined longitudinal outcome, but they should not appear as if they were available at the original decision. Independent evaluation across sites, devices, age groups, and examiners is needed to assess transportability.

### 7.2. Showing What Each Additional Input Contributes

Matched comparisons should begin with a clinically relevant baseline, such as history and otoscopy, while an image-only analysis can quantify the contribution of nonvisual information. Investigators can then add tympanometry and available hearing data. Each comparison should use the same patients and target. Removing one input from the complete model can further show which information contributes to its performance. These analyses should include clinically uncertain cases and should not be restricted to encounters with complete data.

Additional testing also has a cost. Not every patient needs every modality for every clinical question. Studies should examine whether a measurement changes the assessment enough to justify the examination time and patient burden. A rule that selects between already acquired test results is different from a clinical strategy that decides whether to obtain a test. The latter requires prospective evaluation with a prespecified rule and documentation of missed disease as well as avoided testing.

Future models should explicitly represent which inputs are available. A modality mask can distinguish a missing tympanogram from a normal result. Modality dropout, in which selected inputs are withheld during training, may prepare a model for incomplete encounters. Gated feature fusion or late fusion can combine only the available branches, provided that the model is trained and tested for those combinations. These are proposed computational options, not methods already validated for uncertain AOM and OME in the studies reviewed here.

Missingness may depend on age, cooperation, disease severity, or access to equipment. Random modality dropout alone may therefore fail to reproduce clinical practice. If an unavailable measurement is estimated by imputation, the output must identify it as an estimate and preserve its uncertainty. It should not report an estimated hearing threshold as a measured result. Evaluation should include naturally occurring patterns of missing data and report accuracy, calibration, coverage, and withheld diagnoses for each pattern. Inadequate images may still require repeat examination or referral. Excluding all incomplete encounters would recreate the selection problem that the proposed framework seeks to address.

### 7.3. Demonstrating Benefit to Clinical Decisions

Diagnostic studies should report performance for each disease, confidence intervals, and the frequency of uncertain outputs. When a model gives probabilities, studies should also assess whether those probabilities agree with the observed outcomes. STARD-AI and TRIPOD + AI provide relevant reporting guidance [22,23]. Table 4 lists further questions for the clinical problem discussed here. These proposed priorities are not a validated assessment tool.

An image-based reader study provides an example of this type of evaluation. Byun et al. asked 10 otolaryngology residents to classify 71 selected tympanic membrane images before and after viewing the model’s prediction. Mean diagnostic accuracy increased from 82.9% to 90.0% with assistance [24]. This supports a possible assistive role, although the selected-image task did not evaluate treatment decisions or patient outcomes and did not include AOM.

Clinical studies should compare how clinicians assess the same kinds of encounters with and without assistance. A system may improve agreement with a reference yet increase unnecessary examinations or inappropriate treatment. Early evaluation should therefore record clinician responses, usability, and errors introduced by the AI. DECIDE-AI and CONSORT-AI can guide the reporting of early clinical evaluation and subsequent trials [25,26]. Benefits should be defined in terms of appropriate care, with safety outcomes assessed alongside changes in testing or prescribing.

**Table 4:** Questions for evaluating clinical usefulness in uncertain otitis media.

Question	Suggested Evaluation
Does the study include difficult patients?	Report exclusions and retain borderline, resolving, discordant, and technically unsuccessful encounters in the clinical evaluation.
What supports the reference diagnosis?	Specify criteria, reviewer information, disagreement, and uncertainty; report overlap with model inputs.
Does additional information improve assessment?	Compare the same patients and targets with and without each added input; report results in a predefined uncertain subgroup.
Can the system handle incomplete data?	Measure performance and indeterminate outputs when symptoms, acoustic testing, or hearing results are missing.
Is the explanation faithful to the record?	Check each statement against supplied observations; count invented findings and failures to acknowledge uncertainty.
Does assistance improve clinical judgment?	Evaluate appropriate decisions, errors, examination burden, clinician responses, and safety in prospective comparisons.

Proposed priorities for this clinical framework. Established reporting guidance remains applicable [22,23,25,26].

## 8. Discussion

This review proposes that the clinical need for multimodal AI in otitis media is most clearly seen in patients whose findings do not support a confident diagnosis from an image alone. Typical AOM and OME can often be recognized visually. The unresolved problem concerns the patient with subtle or changing morphology, incomplete symptoms, or discordant functional findings. If these encounters are underrepresented during development, good classification performance may not translate into useful assistance at the point of uncertainty.

The studies reviewed here show that images can be combined with acoustic measurements, hearing data, or patient information. Several comparisons report numerical improvements, but their clinical implications differ. Some address broad abnormality detection; others use different class definitions or a reference that shares the added input. Anomaly detection and prediction of diagnostic difficulty also show that uncertainty can be studied without requiring every encounter to fit a simple disease category. These results support further investigation. They do not establish that an integrated system improves decisions in consecutive patients with ambiguous AOM or OME.

The proposed framework also changes what the model should communicate. A clinical encounter contains observations that remain useful even when the final label is uncertain. Evidence of effusion, a conductive hearing deficit, or a discrepancy between symptoms and appearance can be reported without implying that every aspect of the diagnosis has been settled. A readable account of these relationships may be more useful than a categorical answer alone. This should be tested against simpler alternatives, including a structured summary without an LLM, so that any benefit of language generation is demonstrated rather than assumed.

Practical use also depends on the availability of linked data and equipment. Images, history, acoustic results, and hearing tests must be linked to the correct ear and visit. Data collection and transfer require appropriate consent and security. Staff need training to obtain clear otoscopic images. Tympanometry requires a suitable probe fit, maintained equipment, and additional time. Hearing tests also require calibrated equipment and appropriate test conditions [2,18].

In settings with limited resources, a system may need to start with history and otoscopy and accept additional tests when available and clinically indicated. Missing information and uncertainty should remain visible. Offline processing or secure review at another site may be useful when internet access is limited. Studies should measure failed examinations, staff time, cost, and completion of referral. The remote-community study supports research in this setting, but does not establish cost-effectiveness [20].

This review has several limitations. It is a focused narrative review rather than a systematic review. The literature search used PubMed and supplementary source checking, although the completeness of the identified literature cannot be assured. The included studies differed in their patient populations, diagnostic targets, and methods used to establish the reference diagnosis, limiting direct comparison of their results. The evidence was synthesized narratively, without meta-analysis or a formal risk-of-bias assessment. Finally, the proposed framework has not been clinically validated. Future studies should determine whether combining multiple sources of information and providing an explanation improves clinical decisions compared with simpler approaches.

The immediate research priority is a clinically representative paired dataset that retains uncertainty. It should link the image to the history, functional tests, available hearing measurements, and the timing of each observation. Evaluations should determine whether the added information improves assessment in the difficult cases that motivated its collection. This would provide a direct test of whether multimodal AI reduces the gap between selected training examples and routine clinical practice.

## 9. Conclusions

Multimodal AI may help assess patients whose otoscopic findings are incomplete, overlapping, or changing by combining images with symptoms, tympanometry, and hearing information when available. Existing studies support feasibility but provide limited evidence of benefit in uncertain AOM and OME. Future models should retain these encounters during development and evaluation, preserve uncertainty, and describe the current ear status alongside a diagnostic label. LLMs may assist this communication, but accurate and clinically useful explanations require their own validation. The central test is whether the system improves clinical judgment in the patients who are difficult to classify.

## Figures and Tables

**Table 1:** Clinical information that can complement an otoscopic image.

Information	Clinical Question	Main Limitation
Otoscopy or video	What membrane findings are visible, and is the view adequate?	An image cannot establish symptom timing or hearing thresholds.
Symptoms and history	Are the findings associated with an acute episode, persistent symptoms, or recovery?	Missing information is not a negative finding; symptoms may be nonspecific.
Tympanometry	Do functional findings support the suspected middle ear abnormality?	Interpret with tracing quality and ear canal volume; it does not alone establish AOM.
Wideband measurements	What frequency-dependent mechanical changes are present?	Results depend on age, acquisition conditions, and the diagnostic target.
Hearing assessment when feasible	What is the functional hearing impact?	Additional characterization; not required for every encounter or sufficient for AOM–OME differentiation.

AOM, acute otitis media; OME, otitis media with effusion.

**Table 2:** Selected studies combining otoscopic images with additional patient information.

Study and Inputs	Data and Approach	Reported Result	Clinical Interpretation
Binol 2020 [19] Otoscopy and tympanometry	73 paired records; model-development conference report; late voting fusion; age and site count require clarification; independent external validation not established	Accuracy: 74.0% images; 84.9% combined	Higher accuracy for detecting abnormal ears; small conference study; no established external validation.
Stephens 2025 [20] Otoscopy and tympanometry	813 school-aged children, 10 remote South Australian communities; 15,057 matched image samples; retrospective analysis; internal 70/30 split	Accuracy: 78% otoscopy; 82% combined	Image predictions combined with tympanometry. Normal/abnormal target. Repeated frames are not independent children; the split by patient and ear needs clarification.
Sundgaard 2024 [12] Otoscopy and WBT	1014 paired observations; ages 2 months–12 years; one Japanese clinic; intermediate feature fusion; five-fold internal evaluation	Accuracy: 85% images; 86% combined; AOM F1: 0.77 versus 0.82	No-effusion/AOM/OME task. Accuracy gain not statistically significant; reference diagnosis also used WBT; no independent external cohort.
Lu 2026 [13] Video and tympanometry	104 ears from 76 adults at one external site; retrospective multicenter image development; confidence-guided decision fusion; video/ear-level evaluation	Accuracy: 63.46% video; 81.73% combined	Six categories without AOM. External paired evaluation is a strength; best threshold chosen in the test cohort; prospective confirmation needed.
Kim 2023 [8] Images and PTA	1480 paired records; ages 0–80 years; Korea University Ansan Hospital; retrospective development; intermediate fusion with attention and gating; five-fold internal evaluation	Best accuracy 92.9%; five-fold mean 89.7%	Five classes including normal membrane with hearing loss; no AOM/OME. Image-only comparator used four classes; the added diagnostic benefit cannot be determined.
Noda 2024 [9] Images and patient text	Retrospective two-institution Japanese study; 40 prompt-development and 190 validation images; one image/patient; age distribution requires clarification; pretrained vision-language prompting	Accuracy: 82.1%	AOM, OME, cholesteatoma, COM; no normal class. Held-out images do not establish external site validation; no matched test with and without patient information.
Song 2025 [11] Images and PTA	Single center, Korea; 1470 paired records; age not reported. Train: 271 normal; test: 68 normal + 1131 abnormal. Cross-attention feature fusion; normal-data anomaly detection; internal evaluation.	Combined: 96.75% accuracy; reported AUC 0.93. Image-only and PTA-only ablations: each 5.67%, AUC 0.50; all predicted normal.	Includes coexisting disorders, uncertain diagnoses and hearing loss with normal appearance. Failed single-input models limit incremental inference; no external or AOM–OME validation.

**Table 3:** Reference diagnosis and evaluation units in the selected multimodal studies.

Study	Reference Process and Shared Information	Unit and Unresolved Methodological Details
Binol [19]	Abnormality classification; the reference adjudication process requires clarification.	Paired record; age range, center count, assessor blinding and disagreement rules require clarification.
Stephens [20]	ENT-assigned diagnoses using Browning criteria, collapsed to normal/abnormal.	Image sample from video, nested within ears/children; it was not clear which test results assessors could see or how disagreements were resolved.
Sundgaard [12]	Experienced ENT used history, symptoms, otoscopy and WBT; WBT is shared with model inputs.	Paired observation; reference not blinded to WBT; additional reader assessments describe difficulty, not an independent reference for all cases.
Lu [13]	At least two clinicians read video, blinded to tympanometry and AI; consensus for disagreements.	Ear examination/video. Cases without consensus were excluded during development. All 104 assigned paired tests were retained. Visual reference remains distinct from a complete clinical diagnosis.
Kim [8]	Disease and hearing categories; hearing-loss category depends on audiometry.	Paired image/PTA record; the information available to assessors and the way disagreements were resolved need clarification. PTA cannot be assumed to be an independent reference.
Noda [9]	Treating otolaryngologists assigned final diagnoses; clinical context accompanies images.	One image per patient; pooled two-institution data. The information available to assessors and rules for disagreement were not clearly reported.
Song [11]	Normal and six abnormal groups are listed; hearing loss with normal membrane appearance is an abnormal category. Assessor number, diagnostic adjudication and blinding are not reported.	PTA contributes to the target definition as well as the input. Record-level split is reported; patient/ear independence is unclear. No external validation or separate AOM–OME result.

## Data Availability

No new patient-level data were generated or analyzed. Sources discussed in this review are identified in the reference list.

## Abbreviations

Abbreviation	Meaning
AI	Artificial intelligence
AOM	Acute otitis media
OME	Otitis media with effusion
WAI	Wideband acoustic immittance
WBT	Wideband tympanometry
PTA	Pure-tone audiometry
LLM	Large language model

## Appendix A. Literature Identification and Selection

### Appendix A.1. Scope and Sources

A targeted PubMed search was performed on 1 October 2026. Additional source checking was conducted through relevant journal websites and institutional repositories to identify and verify eligible studies.

### Appendix A.2. Search Date and Limits

The PubMed publication-date interval was 1 January 2000 through 1 October 2026, with no language or article-type filter. The lower bound imposed no practical restriction on this topic. The query used title/abstract fields. All 39 returned PMIDs were retrieved; there were no duplicate PMIDs. The exact query and the publication-date filter are shown below.

### Appendix A.3. Exact PubMed Query

(“otitis media”[tiab] OR “middle ear”[tiab] OR “tympanic membrane”[tiab] OR otoscopic[tiab]) AND (“artificial intelligence”[tiab] OR “machine learning”[tiab] OR “deep learning”[tiab] OR “neural network”[tiab] OR “large language models”[tiab] OR GPT[tiab]) AND (multimodal[tiab] OR “multi-modal”[tiab] OR fusion[tiab] OR tympanometry[tiab] OR immittance[tiab] OR audiometry[tiab] OR “patient information”[tiab] OR “clinical information”[tiab]) AND (“1 January 2000”[Date-Publication]: “1 October 2026”[Date-Publication])

### Appendix A.4. Eligibility and Screening

Core eligibility required an original human study combining otoscopy images or video with an additional patient-level functional measurement or clinical input and reporting an evaluable diagnostic result. Studies of other middle ear disorders were retained for their methodological relevance and identified separately from AOM–OME evidence. Reviews, correction notices, nonclinical studies, studies without otoscopic input, image-only classifiers, and studies using hearing tests only as an outcome were outside the core set. A generic text instruction to an image model was not considered additional patient information. Broad ENT studies without separately interpretable middle ear results were also outside the core comparison. Conference studies were eligible when they met the same criteria.

A total of seven relevant studies were identified through the targeted PubMed search and supplementary source checking: Noda, Sundgaard, Stephens, Lu, Song, Binol, and Kim [8,9,11,12,13,19,20]. These studies were summarized in Table 2.

### Appendix A.5. Data Extraction

The included studies were compared according to study design, population, sample size, diagnostic targets, input modalities, reference standards, and validation methods. Attention was given to distinguishing patients from images or repeated measurements and to identifying studies that directly compared image-only and multimodal approaches.
